# Reliability of the chronic mild stress model of depression: A user survey

**DOI:** 10.1016/j.ynstr.2016.08.001

**Published:** 2016-08-22

**Authors:** Paul Willner

**Affiliations:** Dept. of Psychology, Swansea University, Swansea SA2 8PP, UK

**Keywords:** Depression, Chronic mild stress, Reproducibility, Reliability, Sucrose intake, Rat, Mouse

## Abstract

The chronic mild stress (CMS) model of depression is considered by many to be the animal model of depression that has the greatest validity and translational potential, but it has often been criticized for a perceived lack of reliability. The aims of this study were to establish the extent to which the procedure is reproducible, and to identify experimental variables relevant to its reliability. Because failures to replicate frequently remain unpublished, a survey methodology was used. A questionnaire was circulated to 170 labs identified from a PubMed search as having published a CMS study in the years 2010 or 2015 (with no selection in respect of the results reported). Responses were returned by 71 (42%) of the recipients, followed by further correspondence with some of them. Most of the respondents (n = 53: 75%) reported that the CMS procedure worked reliably in their hands. Of the others, 15 (21%) reported that the procedure was usually reliable, but not always (n = 9: 13%) or not for all measures (n = 6: 8%). Only three respondents (4%) reported being unable to reproduce the characteristic effects, two of whom may be using an insufficient duration of CMS exposure. A series of analyses compared the 75% of ‘reliable’ labs with the 25% of ‘less reliable’ labs on a range of experimenter, subject, stress and outcome variables. Few if any significant differences between these two samples were identified, possibly because of the small size and diversity of the ‘less reliable’ sample. Two other limitations of the study include the (unavoidable) omission of labs that may have worked with the model but not published their data, and the use of ad hoc measures to compare the severity of different stress regimes. The results are discussed in relation to relevant published observations. It is concluded that CMS is in fact a rather robust model, but the factors that result in a less effective implementation in a minority of laboratories remain to be firmly established.

## 1 Introduction

Chronic mild stress (CMS) is a well-validated and widely used animal model of depression, based on the loss of responsiveness to rewards by animals subjected to a varying schedule of minor stressors. The CMS model was developed in the late 1980s, on the basis of an earlier observation that rats subjected to a variety of relatively severe stressors failed to increase their fluid intake when sucrose or saccharin was added to their drinking water (Katz, 1982). The aims of the early CMS work were: to engender similar effects using a much more mild and ecologically valid stress regime; to explore the concept of stress-induced anhedonia by investigating the effects of CMS on a variety of reward-related behavioural endpoints; and to confirm the utility of the model as a test-bed in which to investigate the mechanisms of action of antidepressant drugs (Willner et al., 1987, Willner et al., 1992). The CMS procedure was implemented by exposing rats (or later, mice: Monleon et al., 1994) to a relatively continuous variety of mild stressors, such as periods of food and water deprivation, changes of cage mates, and other similarly innocuous manipulations. Over a period of weeks of chronic exposure the animals gradually reduced their consumption of, and preference for, a preferred dilute sucrose solution, and this deficit could be reversed by chronic, but not acute, treatment with antidepressant drugs. The development and validation of the CMS model are described in more detail in earlier reviews, and in the accompanying paper (Willner, 1997a, Willner, 2005, Willner, 2016).

As the CMS model was taken up by other labs in the early 1990s, concerns began to emerge about the reproducibility of the effects reported. Inter alia, this concern was highlighted by the fact that the procedure became less reliable in the hands of the original research group following a move to a different university. These issues, and others, were debated in detail in a Special Issue of the journal Psychopharmacology, which included a candid account of the first decade of CMS research (Willner, 1997a) and sixteen peer commentaries. The response to the peer commentaries summarized the position regarding the reliability of the CMS model as follows: “some laboratories, including, currently, our own, have experienced difficulty in (re)establishing the CMS procedure, but there are many other laboratories in which the procedure operates reliably” (Willner, 1997b). A later review summarized data from over a hundred labs reporting depressive-like (and antidepressant-reversible) effects of CMS across a wide range of depression-relevant end-points, including sucrose or saccharine intake or preference, sweet food intake, approach to sweet food, place conditioning using a variety of drug and natural reinforcers, brain stimulation reward, immobility in the forced swim test, learned helplessness, male aggression and sexual behaviour, grooming, and REM sleep latency (Willner, 2005). However, that review also identified a handful of studies, including several published only as meeting abstracts, reported ‘anomalous’ effects of CMS, such as increased sucrose intake or brain stimulation reward (Willner, 2005).

Partly as a result of the uncertainty described in the 1997 and 2005 review papers, there has been a frequently expressed assumption that the CMS procedure is unreliable or difficult to replicate, and reviews of animal models of depression typically include a statement to this effect. However, this conclusion does not sit comfortably alongside the burgeoning CMS literature, which, as described in the accompanying paper, now amounts to in excess of 1300 publications, that in the year 2015 alone include 230 papers from 180 labs in 30 countries (Willner, 2016). These statistics, and the exponentially increasing uptake of the CMS model (Willner, 2016), suggest that the model may be more reliable than is typically assumed. The aims of the present study were to attempt to quantify the extent to which the CMS model is reliable and to understand some of the relevant factors. The main focus was on the reliability with which CMS elicits the most widely used outcome, a decrease in sucrose intake or preference.

Investigating the reliability of an experimental procedure presents particular problems because of the possibility that the published literature represents the tip of an iceberg, with failures to replicate and other evidence of unreliability lying below the surface, unpublished. In order to take account of this issue, the present study adopted a survey methodology in preference to a systematic literature review, on the assumption that asking users about their experience of working with the CMS procedure would be more likely to yield insights into the problems they might have encountered. Another methodological issue that needed to be addressed at the outset is that different labs refer in different ways to procedures that may be similar or may diverge: alongside CMS, other labels include chronic unpredictable stress (CUS) and unpredictable chronic stress (UCS), chronic unpredictable mild stress (CUMS) and unpredictable chronic mild stress (UCMS), and chronic varied or variate stress (CVS). It was decided to take an inclusive approach to the survey, and this decision was vindicated by the outcome: an analysis presented in the accompanying paper (Willner, 2016), shows that empirically – and perhaps surprisingly – these different labels carry no information about the severity of the stress protocol and almost no information about the predictability of stress. In this paper, therefore, CMS is used as a generic term covering all of these procedures.

## 2 Methods

### 2.1 Survey methodology

An initial PubMed search using the search terms [chronic (mild or varied or unpredictable) stress] returned over 3000 hits. In order to narrow this literature down, the search was repeated for single years at 5-year intervals from 1990 to 2015 (with the final search on December 31, 2015), and the outputs were searched by hand to identify papers involving varied stress regimes in animals. Studies in people and animal studies involving repeated presentation of a single stressor were excluded. This search indicated an exponential increase in publications, rising above 100 in 2010. The years 2010 and 2015 were chosen for further investigation, on the basis that authors publishing in 2015 had recent experience with the CMS methodology, while those publishing in 2010 might have encountered difficulties that had caused them to cease working with the model, but should still have a good memory of their experiences. Papers from 2010 and 2015 were ordered by country and region, in order to identify independent laboratories, and email addresses were collected where easily available from PubMed abstracts or open access publications, supplemented in a few cases by addresses already known to the author.

Each of the labs for which an email address was identified was sent a survey, created using Google Forms, and asked to return it via a web link. A total of three further requests were made to non-responders. Following receipt of an email explaining that Google was not readily available in China, the second and third requests to Chinese recipients invited them to return the survey via email; this offer was also extended to other respondents at the third request. The survey covered the basics of the methodology used, followed by sections probing within-experiment reliability and between-experiment reliability. The survey is not presented in detail because many of the questions returned indeterminate answers, such as a high proportion of missing or ambiguous responses. Details of the questions for which responses could usefully be analyzed are presented in the Results section.

Subsequently, follow-up questionnaires were emailed (i) to respondents who indicated that in their lab the procedure was “usually reliable but not always”, to probe the nature of unreliable performance and potential differences between more and less successful experiments, and (ii) to respondents who indicated that they did not use a sucrose intake or preference test. Again, details of the questions asked are presented in the Results section.

### 2.2 Estimation of CMS intensity

In order to compare the severity of different stress regimes a two-stage Delphi procedure was used to obtain ratings from five experts with extensive use of the CMS procedure. A list of 26 micro-stressors was compiled from responses to the survey, each of which was rated independently by the five raters, using a 5-point scale of severity. The ratings were then shared, anonymously, with the other raters, together with a few comments made on the first round. The ratings were then repeated, this time with separate ratings for rats and mice. Kendall's coefficient of concordance was used to assess the degree of agreement between the five raters. Concordance was relatively low on the first round (W = 0.45, p < 0.001), and increased somewhat on the second round but remained below the minimum acceptable level of 0.6 (rats: W = 0.53; mice: W = 0.59). Considering that the raters included two rat experts and two mouse experts, plus the author, the concordance was calculated for three raters for each species (the two relevant experts plus the author). Both analyses achieved concordances of W = 0.75 (p < 0.001). The median of these three ratings for each micro-stressor and species was then used to construct three measures to characterize the different stress regimes: Variety (the number of elements included), Severity (the proportion of elements rated as 4 or 5) and the overall Burden of stress (the sum of the ratings across all of the micro-stressors identified). The Burden measure in particular is a very rough and ready estimate that does not take account of the frequency or duration of each element, that the severity of individual elements may vary markedly between laboratories, or that combinations of stressors are sometimes applied; this caveat also applies, to a lesser extent, to the Variety and Severity measures.

## 3 Results and discussion

A total of 245 labs were identified as publishing in 2010 (n = 81) and 2015 (n = 181), with a surprisingly low proportion (6.5%) represented in both years (Table 1). A total of 170 survey requests were mailed out, representing 70% overall of the labs identified as publishing in those years. A total of 71 responses were received, representing a 42% return overall, with a relatively low return (17%) from China but a 60% return from other regions. This was a relatively experienced sample: while fourteen respondent had performed only one (n = 8) or two (n = 6) CMS experiments, many had conducted 3–5 experiments (n = 15), and the majority (n = 41) had completed more than 5 experiments. As some of the respondents asked for their responses to be treated anonymously, none of the respondents have been identified by name. (For audit purposes, respondents are identified by their location in the database.)

### 3.1 Overall reliability of the CMS procedure

Only three of the 71 respondents reported that they do not typically see a depressive phenotype. One of these respondents (R11) had nonetheless published at least five papers reporting anhedonic-like effects of CMS. The other two (R31, R38) used very brief (10 and 14 days) periods of CMS exposure, compared with a modal duration of stress exposure among the other respondents of 3–4 weeks, with 70% of respondents (37/53) reporting that more than 2 weeks of stress exposure was needed to see an effect of CMS, as documented in literally hundreds of publications.

Initially, twenty four respondents (34%) reported that CMS was “usually but not always” reliable. However, nine of these clarified that what they meant by this was that “Effects of stress were always significant but some individual animals failed to respond”. This left 15 respondents (21%) reporting genuine unreliability (in addition to the three respondents who reported a generally negative experience). However, six of these respondents (R1, 32, 46, 63, 68, 69) reported that “Stress did not reliably decrease sucrose intake/preference but did reliably elicit other depression-like effects”. Of the other nine respondents, three (R5, 48, 57) reported that “Effects of stress were present in every experiment, but not always statistically significant”, one (R26) reported that CMS was effective in one rat strain but not in another, and only five respondents (R13, 22, 32, 42, 49) endorsed the statement that “In some experiments stress had no apparent effect”.

A possible under-estimate of unreliability arises from the fact that some respondents (n = 16) reported that they did not use a sucrose intake/preference test, raising the possibility that the reason was that they had tried it and found it unreliable. A follow-up query to these respondents elicited 14 replies. Nine respondents said that they had never tried the sucrose test, two said that they had used the sucrose test and found it to be reliable, but now preferred to use other measures, and one (R29) reported using a sweet food test in preference. Only two of these respondents said that they did not use the sucrose test because it did not work reliably, and one of these (R54) explained that this was because of excessive spillage from water bottles. Only a single respondent (R1) reported not using the sucrose test because of unreliability of CMS effects, and as already reported above, this respondent did see reliable effects on other behavioural measures.

Overall, therefore, a total of twelve respondents (17%) reported that CMS sometimes failed to produce depressive-like effects (nine of whom nonetheless reported that they found CMS to be “usually reliable”), with a further six respondents (8%) reporting unreliability of the sucrose intake/preference test but a reliable response on other measures. As described below, a series of further analyses was carried out comparing these 18 respondents with the other 53 (75%), in relation to experimenter, subject, stress, and outcome variables. An initial analysis compared the experience of the two groups. There was a tendency for labs in the ‘less reliable’ group to have conducted more CMS experiments than those in the ‘reliable’ group, which is to be expected: the more experiments undertaken, the greater the likelihood of seeing an occasional failure. However, the difference is not statistically significant (Fisher's exact test: proportion of labs conducting >5 experiments, 72% vs 54%, p = 0.267; proportion conducting >2 experiments, 94% vs 75%, p = 0.096).

Reports of the overall reliability of the CMS procedure did not map as clearly as one might wish onto another estimate of reliability, the proportion of animals responding to CMS. As shown in Table 2, while 27 (56%) of the labs reporting reliable effects of CMS estimated that >75% of animals responded, 7 of them (15%) estimated a relatively low response rate (<50%). Nevertheless, the proportion of animals responding to CMS was significantly greater overall in labs reporting that CMS produced a reliable depressive-like phenotype (Chi-square = 8.0, p < 0.02, after amalgamating the two ‘less reliable’ groups). This is despite the fact that only around a third of the respondents were able to specify the criterion they used to define an individual ‘responder’, and these criteria varied considerably. Overall, 85% of respondents reported that >50% of animals responded to CMS, or 80% if the three labs that reported not seeing a depressive phenotype are included in the calculation.

Significantly, the three respondents who reported not seeing a depression-like phenotype all said that they see no effect of CMS on prototypical depression-like parameters, rather than an effect in the ‘wrong’ direction (such as increased sucrose intake/preference or decreased immobility in the forced swim test). That is, none of the 71 respondents to this survey reported seeing the ‘anomalous’ effects of CMS that are sometimes reported (Willner, 2005), suggesting that these effects are rather rare.

### 3.2 Experimenter variables

Two of the respondents (R1, 9) who reported not seeing reliable effects of CMS on sucrose intake reported that they nonetheless had at one time had a worker in their lab who did reliably see this effect, which they were unable to explain. Another respondent (R63) commented that “untrained personnel in our animal care facility will wreak havoc with the protocols”, which provides the basis for one potential explanation.

Experimenter effects have been previously described in relation to other behavioural paradigms (Crabbe et al., 1999), with the suggestion that “Different experimenters … probably presented idiosyncratic arrays of odor cues and handled the mice somewhat differently” (Wahlsten et al., 2003). The potential effect of odour cues in the present context was examined in two ways. A first question was whether lab personnel were allowed to wear perfumes or deodorants. The proportions of labs that forbade these sources of odour cues were 9/18 (50%) in the ‘less reliable’ group of labs, and 29/53 (55%) in the ‘reliable’ group: essentially, the same. A second question was the direction of air flow: whether pheromones were transmitted from animals to people or from people to animals. (This was a potentially significant factor in the author's own experience: the loss of CMS reliability coincided with a change of animal house from one with animals-to-people airflow to one with people-to-animals airflow.) The proportions of animal houses exposing animals to human pheromones from people to animals was 2/13 (15%) in the ‘less reliable’ group of labs and 6/32 (19%) in the ‘reliable’ group of labs: again, essentially the same. Despite these negative results, one respondent (R2) pointed out that, given rodents' extreme olfactory sensitivity, it is very likely that their behaviour could be influenced by odorants such as perfumes, deodorants or detergents, and therefore these potential sources of error should be proscribed.

### 3.3 Subject variables

An initial examination of species used found that the group of 18 ‘less reliable’ labs included 5 working with mice, 9 working with rats and 4 working with both species, compared with 25, 26 and 2 labs in the ‘reliable’ group. Notably, however, 40% of mouse labs (the same proportion among the ‘less reliable’ and ‘reliable’ groups) did not use the sucrose intake test, compared with only 9% of the rat labs (Fisher exact test, p < 0.005). The fact that the sucrose test is used more frequently in rats than in mice raises the possibility that the test may work better in rats, as suggested by some respondents (notwithstanding that when asked, most of the non-users reported that the decision not to use the sucrose test was not based on their own experience). If this is the case, it could reflect either a differential effect of CMS or, perhaps more likely, the greater difficulty of measuring fluid intakes accurately in smaller animals.

The major strains used, across labs, were Sprague Dawley (SD) and Wistar rats and C57BL/6 and BALB/c mice. SD and Wistar rats were used, respectively, by 4 and 4 labs in the ‘less reliable’ group and by 10 and 12 labs in the ‘reliable’ group, suggesting no difference in susceptibility between these two rat strains. The two mouse strains were used, respectively, by 3 and 3 labs in the ‘less reliable’ group of labs and by 3 (BALB/c) and 16 (C57BL/6) labs in the ‘reliable’ group; this difference, while suggestive, is not significant (p > 0.1). Indeed, while C57BL/6 mice are used more frequently, two respondents (R31, 41) suggested that they are actually less susceptible to CMS than BALB/c mice. This would be consistent with published studies reporting that BALB/c mice are more susceptible than C57BL/6 mice to CMS (Griffiths et al., 1998; Ducottet and Belzung, 2004, Ducottet and Belzung, 2005) and in other stress paradigms (Anisman et al., 1998, Anisman et al., 2001, Razzoli et al., 2011a, Razzoli et al., 2011b, Savignac et al., 2011). (Although C57BL/6 mice are not the most resistant strain: in a different comparison DBA/2 mice were more resistant than C57BL/6 mice to CMS effects on sucrose intake, with CBA/H mice the most susceptible among the strains tested: Pothion et al., 2004.) There are several other reports in the literature of strain differences in sensitivity to CMS, with increased responsiveness reported for Flinders Sensitive Line rats (Pucilowski et al., 1993), and for several genetically modified mouse strains (CB1 knockout: Valverde and Torrens, 2012; OCT2 null mutant: Couroussé et al., 2014; VGLUT1+/−: Garcia-Garcia et al., 2009).

Sex was mentioned by 12 respondents as a critical factor determining the effects of CMS in their hands, but generally without specifying the direction of effect, and with mixed findings reported by those who did provide more detail. Recent systematic reviews of sex effects in CMS report a similar mixed picture (Dalla et al., 2010, Franceschelli et al., 2014). Four respondents mentioned age as a factor, with greater susceptibility to CMS in middle-aged rats relative to younger animals. There is some support in the literature for this observation (Herrera-Pérez et al., 2008, Toth et al., 2008). A greater response in middle-aged rats relative to older animals was also mentioned (R43, 49, and Pardon et al., 1994). There are other isolated reports of effects of factors such as rearing conditions, endocrine status and nutritive status (Willner, 2005, Table 7). Finally, one respondent (R57) reported the experience from three separate labs of a difference in susceptibility to CMS in rats of the same strain from different US suppliers. A recent paper reported differences in susceptibility to CMS among outbred Wistar rats from three different European suppliers (Theilmann et al., 2016) and similar effects have been documented in other behavioural paradigms (e.g. Palm et al., 2012, Goepfrich et al., 2013, Browne et al., 2015).

### 3.4 Stress variables

As noted earlier, two of the three respondents who did not typically see a depression-like phenotype following CMS used a relatively short exposure (<2 weeks). However, as shown in Table 3A, the overall duration (days) of stress exposure was (non-significantly) longer in the labs reporting ‘less reliable’ effects. Nevertheless, three respondents in the ‘less reliable’ group (R26, 46, 63) stated that in their experience the duration of stress needed to be at least six weeks, consistent with a published report that mice of the relatively stress-insensitive C57BL/6 strain displayed a reliable depressive-like phenotype after an 8-week CMS regime but not after 4 weeks of CMS (Monteiro et al., 2015). Table 3A also compares the two reliability groups on the three measures of CMS intensity: Variety, Severity and Burden. Again, no notable differences were seen. The same variables were examined in relation to the proportions of animals responding to CMS (Table 3B). Again, there were no differences on any of the four stress variables. Table 3 reports combined data for rats and mice, but the picture is unchanged if the two species are examined separately.

The most frequently used micro-stressors are listed in Table 4. There were significant or near-significant differences in the use of certain micro-stressors in mice and rats: in particular, food and water deprivation are less frequently used with mice than with rats. However, for no individual micro-stressor was there a significant difference in use between ‘reliable’ and ‘less reliable’ labs, or between labs reporting different proportions of animals responding to CMS (cf. Table 3). Nevertheless, seven respondents commented that they had modified the procedure either to increase the intensity of the CMS regime or to reinstate certain micro-stressors that led to a decrease in efficacy of CMS when removed. Examples included replacing cage tilt with exposure to predator odour (R49) or presenting two micro-stressors simultaneously (R45). Just over half of the respondents (57%) said that their procedure was based on the original CMS publications (Willner et al., 1987, Willner et al., 1992), with others citing more recent studies from other labs. However, when taking into account both composition (micro-stressors included) and delivery (fixed/random) it was not possible to identify any two CMS schedules that were identical, either for rats or mice.

The use of food and water deprivation by most labs is relevant to the question of whether the effect of CMS on sucrose intake is influenced by changes in body weight. This was suggested as the decisive factor in early work from one lab which reported that the effect of CMS on sucrose intake was non-significant when expressed in relation to body weight, which increases more slowly, or may even decrease, during exposure to CMS (Matthews et al., 1995, Forbes et al., 1996). Many other studies, however, demonstrated that CMS was effective even after correcting for changes in body weight (reviewed by Willner, 1997a; see Papp et al., 2016 for a recent example). Of note, eight of the present respondents reported using sucrose intake/body weight as their primary outcome measure, and seven of these (R2, 8, 10, 12, 37, 50, 58) reported reliable effects of CMS. Another significant aspect of the present data is that while around two thirds of labs do use food and water deprivation, around one third of labs do not, and CMS is equally effective in both cases. Also relevant to this debate is that 11/12 labs in the ‘less reliable’ group and 37/43 labs in the ‘reliable’ group reported using loss of sucrose preference rather than decreased sucrose intake as the outcome measure, which is unrelated to lower body weight. Overall, the present data support the earlier conclusion that CMS effects cannot be explained away as an artefact of metabolic changes.

### 3.5 Outcome variables

Most respondents reported that they conduct the sucrose intake test in the animal's home cage rather than moving it to a test cage, and this variable did not differ between reliable and ‘less reliable’ labs (4/33: 12% vs. 2/12: 17%). However, in relation to the timing of tests there are hints of diurnal and circannual effects. One respondent (R46) reported seeing stronger CMS effects when testing was at the start of the dark phase of the light-dark cycle, as reported in earlier published study (D'Aquila et al., 1997a). But a more recent study using a different experimental design reported a different pattern of diurnal effects (Aslani et al., 2014), and Papp (2012) recommends testing at the start of the light cycle, so there is no consensus on the best time of day to test. Three respondents (R46, 57, 68) indicated that CMS may be less effective in the summer months. Circannual effects have not been formally studied in the CMS model, but there is some evidence for their existence in other stress paradigms (Borsini et al., 1990, Meyer et al., 2006, Kiank et al., 2007, Han et al., 2014).

Two important variables in the sucrose intake/preference test are the extent of food/water deprivation immediately prior to the test and the concentration of the sucrose solution. (The survey did not ask about the duration of the sucrose test but this is typically related to the duration of food/water deprivation, with brief deprivation (0–1 h) usually preceding a prolonged (e.g. overnight or 24 h) test and long deprivation (e.g. 12 h) usually preceding a brief (e.g. 1 h) test). The majority of respondents (41/57: 72%) reported using relatively long periods of deprivation (3–11 h, n = 9; >12 h, n = 32). This was similar between rat and mouse labs, and also between reliable and ‘less reliable’ labs. However, some differences were found in the concentrations of sucrose used in different labs. All but one (R35) of the mouse labs reporting these data (14/15: 93%) used 1% sucrose, but among rat labs, only 18/30 (60%) used 1% sucrose with the other 12 labs using concentrations of 2% or higher (Fisher exact test, p = 0.034). Further examination of the rat data showed that most (16/22: 73%) of the labs reporting reliable data used 1% sucrose, whereas most (6/8: 75%) of the ‘less reliable’ labs used more concentrated (2–5%) sucrose solutions (Fisher exact test, p = 0.034). This suggests (albeit weakly, given the low numbers and the fact that the effect was seen only in rats) that it may be important for CMS reliability in the sucrose test that the sucrose solution is only weakly rewarding.

Some labs do not use the sucrose test (as discussed above) and not all of those that do see it as their primary behavioural endpoint. Table 5 lists the primary outcome measures reported. The table separates out the ‘less reliable’ group of labs into two subgroups: those (group 1: n = 12) reporting that CMS was unreliable (n = 3), or “usually but not always reliable” (n = 9), and those (group 2: n = 6) reporting that CMS “did not reliably decrease sucrose intake/preference but did reliably elicit other depression-like effects”. Unsurprisingly, those labs in which CMS was reported to work reliably for measures other than the sucrose test (group 2) did not use the sucrose test as their primary outcome variable. Two other trends are visible in the data: the labs reporting reliable CMS effects (group 3: n = 53) tended to use a broader range of outcome measures; and none of the labs in group 1 reported using the forced swim test for their primary outcome. However, neither of these trends was statistically significant.

Different labs varied in when outcome tests were performed: 28 labs gave a single test at the end of the CMS procedure; 8 labs administered tests before and after CMS; and 31 labs tracked the effect of CMS at least weekly (typically using a sucrose or coat-state test). Repeated testing makes it possible to establish a pattern of effects, such as a rapid habituation of early CMS effects or an unstable control baseline. (The latter effect, but not the former, could also be detected by testing twice, before and after CMS.) Those respondents who reported that effects of CMS were less than reliable were asked whether either of these patterns was present. None reported seeing early effects of stress that rapidly habituated, as reported in several studies (D'Aquila et al., 1997b, Pothion et al., 2004, Schweizer et al., 2009), but four respondents (R42, 46, 48, 49) reported that, while stress appeared to decrease sucrose intake, the behaviour of the control group also changed, suggesting that a component of unreliability may be that control animals were inadvertently stressed. This could easily occur if controls and CMS animals are housed in the same room, a procedure which should strictly be avoided.

## 4 General discussion

### 4.1 How reliable is the CMS model?

A recent review of animal models of depression offers the following summary: “Chronic mild stress utilises the anhedonic responses as measured by a reduction in consuming a sweet solution (a pleasurable activity) following a series of mild stressors … However, this model has had difficulties in being replicated in all laboratories” (Kelly, 2009). This is a sentiment commonly encountered in the literature, sometimes in a more extreme form. Indeed, according to the Wikipedia article on Animal Models of Depression (2016) “The chronic mild stress (CMS) model is probably the most valid animal model of depression (… but the) data can be hardly replicated”. Similarly, the Encyclopedia of Behavioral Neuroscience states that “(The CMS model) has very poor reliability and CMS-induced effects could not be reproduced in many laboratories … (The) model has fallen out of favour due to the lack of cross-laboratory reliability” (Cryan, 2010). Considering the steady rise in the number of CMS publications, and the fact that the number of labs publishing CMS studies more than doubled from 81 in 2010 to 180 in 2015, the notion that the CMS model “has fallen out of favour” is clearly false. The main aim of the present investigation was to establish what difficulties users of the CMS model actually experience, and whether the accepted notion that the procedure is difficult to replicate stands up to scrutiny.

It is certainly true that in the early years (pre-2000) many labs experienced difficulties in reproducing CMS effects and this gave an impression of unreliability, summarized in an influential review in the following terms “… the behavioral abnormalities produced by chronic stress … have been difficult to replicate across laboratories, which has reduced their general application” (Nestler et al., 2003). Nevertheless, a slightly later review commented that “… while the CMS model can undoubtedly prove difficult to establish … the above conclusion appears to be based on the literature as it existed some years ago, and reveals a lack of familiarity with the explosion of more recent publications” (Willner, 2005). Indeed, the present data, which describe the situation a decade later, demonstrate that the reproducibility of CMS effects is no longer a serious concern.

An overwhelming majority of the respondents to the present survey (n = 53: 75%) reported that the CMS procedure worked reliably in their hands. Of the others, 15 (21%) reported that the procedure was usually though not always reliable, (n = 9: 13%), or that they had difficulty with the sucrose test but not with other measures (n = 6: 8%). Only three respondents (4%) reported being unable to reproduce the characteristic effects, two of whom may be using an insufficient duration of CMS exposure. And even the single respondent who finds the CMS procedure to be unreliable after an adequate trial has actually published a string of papers reporting anhedonic-like effects of CMS. A limitation of the study is that this was not a representative sample. The questionnaire could not be circulated to research groups that may have worked unsuccessfully with the CMS model but not published their data, as such labs could not be identified. However, in relation to the potential respondents identified as having published a CMS study in the two years examined (2010 and 2015), the only selection criterion was that they should have a readily accessible email address: no selection was applied in respect of the results reported. Except for China where the response rate was lower, there was an excellent response rate (60%), and respondents were guaranteed anonymity. So if respondents had experienced difficulties with the CMS model, whether or not these were apparent in their publications, there is a reasonable presumption that this should have been reflected in their responses to the survey. The fact that such reports were minimal does suggest that the reproducibility of CMS effects is very much higher than is often assumed.

### 4.2 Can critical factors be identified?

The second aim of the study, to identify the critical factors in reproducibility of CMS effects was less successful. Very few differences were identified between the 75% of ‘reliable’ labs and the 25% of ‘less reliable’ labs on a wide range of experimenter, subject, stress and outcome variables. This may to some extent reflect the small size and, particularly, the diversity of the ‘less reliable’ sample, which contains at least four very small sub-groups. Therefore, the results of these analyses were presented in the context of the published literature. While few if any definitive conclusions can be drawn, a number of factors are worthy of comment.

Individual differences, in relation to both experimenters and experimental subjects, are clearly of importance even if few general conclusions emerge. It is particularly striking that even the respondent reporting the most negative overall picture (R9) could identify a specific worker who did obtain reliable data over a prolonged period. Differences between experimenters, either intrinsic (e.g. pheromonal) or extrinsic (e.g. training), could be expected to account for some of the individual variability in CMS outcomes, as has been suggested in other behavioural contexts (e.g. Wahlsten et al., 2003), even though the present survey did not identify relevant factors.

Differences between experimental subjects have been better characterized, though again, the relevant data are in the literature rather than the present results. Perhaps the best characterized effect is the differential stress susceptibility of C57BL/6 and BALB/c mice. This difference was not, however, apparent within the present data-set, and while the literature is clear that C57BL/6 mice are more resilient to a variety of stressors than BALB/c mice, the greater use of the former strain by respondents to the survey testifies to the greater popularity of C57BL/6 mice as experimental subjects. While rat strains also differ in their susceptibility to stressors (for example, greater responsiveness in FSL or Wistar-Kyoto strains), the literature does not suggest differential stress responsiveness between the Wistar and Sprague-Dawley strains most commonly used in CMS research.

A related issue, which was touched on only in passing in the survey, is variability within populations of animals of the same strain. It is usually regarded as an inconvenience that there is typically a sub-group of animals that do not respond to CMS. However, two groups have promoted as a virtue the fact that it is possible to optimise the procedure so as to identify subgroups of CMS-susceptible and CMS-resilient rats (Wiborg, 2013) and mice (Strekalova and Steinbusch, 2010), because they can then be used to study the neurobiological mechanisms underlying stress susceptibility and resilience (e.g. Couch et al., 2013, Nieto-Gonzalez et al., 2015). Individual susceptibility to CMS has been related to high levels of anxiety (Ducottet et al., 2004, Ducottet and Belzung, 2005, Li et al., 2010) and to a socially submissive behavioural trait (Strekalova et al., 2004, Strekalova et al., 2011), factors that are rarely measured as baseline variables in CMS experiments. There is also a suggestion that CMS may work best in middle-aged animals, though more data are needed on this point. The potential for individual differences to contribute to variability of outcomes cannot be over-emphasized: for example, Strekalova et al. (2011) observed that, in different studies using C57BL/6 mice, the proportion of socially submissive animals varied between 15 and 85%.

Judging from responses to the survey, the study did not support the view, often expressed, that CMS is more difficult to implement in mice than in rats. However, the sucrose test is used significantly less frequently with mice than with rats. As with some other results reported here, it is not clear to what extent this reflects a genuine species difference in ease of use, or simply a personal preference for using a different test, such as the coat-state (grooming) test (Nollet et al., 2013), which is often used with mice but only rarely with rats. However, it is certainly the case that sucrose intake is more variable, and therefore less accurate, in mice than in rats (Strekalova and Steinbusch, 2010), and some evidence-based guidelines exist for increasing the accuracy of the test in mice, including pre-exposure to sucrose to overcome neophobia, switching bottle positions to overcome side preferences, the use of a prolonged two-bottle preference test, and measures to reduce spillage (Strekalova and Steinbusch, 2010, Strekalova et al., 2011). Guidelines for rat studies suggest that reliability can be improved by screening animals before CMS to exclude those (typically around 20%) with very low, very high or very variable sucrose intakes (Papp, 2012).

One factor that was significantly different between the ‘reliable’ and ‘less reliable’ groups of labs was the sweetness of the sucrose solution, with more concentrated sucrose solutions tending, in rats, to be associated with less reliable results. While this result could represent a type 1 error, considering the overall number of comparisons between the two groups of labs, there is a basis for thinking that it could be genuine. The concentration-intake curve for sucrose is bell-shaped (at least in brief tests in rats), and this means that at higher concentrations, a single-bottle sucrose test, or a two-bottle sucrose/water preference test, does not provide a good measure of hedonic value. When comparing medium and high sucrose concentrations, the stronger solution appears less preferred in a choice between sucrose and water, but in a test offering a choice between two sucrose solutions the stronger solution is always preferred (Phillips et al., 1991, Muscat et al., 1991, Willner et al., 1991). In other words, if the concentration of sucrose is too high, a decrease in its rewarding value may not be expressed as a decrease in intake (or in preference with respect to water). A dissociation between intake and reward is also seen with sweet food: for example, CMS was found to increase rats' intake of very sweet food, as is also seen in depressed people, while at the same time decreasing the rate of eating, consistent with a decrease in reward value (Sampson et al., 1992). A decrease in sucrose preference may be very difficult to detect if preference is similar at the sucrose concentration selected for testing and at lower concentrations (cf. Pothion et al., 2004). The optimal sucrose concentration for testing the anhedonic effect of CMS is one that engenders a clear preference over water, but is on the ascending limb of the concentration-intake curve. This would be expected to vary between strains and species, and should be determined empirically in each case, since problems are to be expected if the sucrose concentration is too high. In line with this reasoning, it is recommended that, when working with rats, “the sucrose solution should not exceed 2%” (Papp, 2012).

Another factor that has been reported to influence the effectiveness of CMS is the severity of the stress regime, as commented by some of the respondents to the survey. The ‘reliable’ and ‘less reliable’ groups of labs did not differ on any of the measures constructed to compare the intensity of different stress regimes, but as already acknowledged, these were crude measures that did not take into account the duration of individual micro-stressors or how exactly they were applied. In one of very few studies to examine the effect of parametric variation of CMS intensity, Strekalova and Steinbusch (2010) reported on the greater efficacy of a more intense CMS regime to decrease sucrose intake in mice, where intensity was defined by features such as the duration or proximity of individual micro-stressors. In an early study, Muscat and Willner (1992) reported that a continuous and varied CMS regime decreased sucrose intake to a greater effect than a subset of micro-stressors presented at night only, and that a subset of ‘proximal’ (in-cage) stressors, such as paired housing (of rats that were normally singly housed) or wet bedding, was able to decrease sucrose intake, while a subset of ‘distal’ (out-of-cage) stressors was not. In these experiments, daily presentation of only one element of the ‘proximal’ set, paired housing, was sufficient to maintain a low level of sucrose intake for up to three weeks. However, this particular social stressor was neither a necessary component of the CMS regime, since combinations of stressors that excluded this element were also effective, nor a sufficient component, since with more prolonged administration, repeated pairing alone in the absence of other stressors led to habituation of the response (Muscat and Willner, 1992). Nevertheless, a more intense social stressor, repeated exposure to social defeat does elicit a sustained decrease in rewarded behaviour in rats (Willner et al., 1995, Von Frijtag et al., 2000, Rygula et al., 2005; Miczek et al., 2011) and mice (Krishnan et al., 2007, Yu et al., 2011; Zhang et al., 2015).

The probable influence of CMS intensity on experimental outcomes raises the question of how this should be assessed. The measures used here were based on subjective estimates of the intensity of the individual micro-stressors, and notwithstanding that the estimates were arrived at through expert consensus, this cannot be considered to be a reliable method, and is not recommended. Loss of body weight relative to control animals may provide a more reliable measure of CMS intensity. In some studies, subgroups of rats (Remus et al., 2015) or mice (Strekalova and Steinbusch, 2010, Tang et al., 2013) that displayed vulnerability to a CMS-induced decrease in responsiveness to rewards also showed a relative loss of body weight, while animals that were resilient to induction of anhedonia did not. The relationship between CMS effects on hedonic parameters and body weight is far from absolute: anhedonic effects are sometimes seen in the absence of an effect on body weight in both rats (e.g. Muscat and Willner, 1992, Papp et al., 1992, Valverde et al., 1997) and mice (Griffiths et al., 1992, Pothion et al., 2004), and the opposite effect, a relative decrease in body weight in the absence of changes in sucrose intake or preference, has also been seen (Gouirand and Matuszewich, 2005, Chang and Grace, 2014). In general, however, CMS studies that report a decrease in sucrose intake or preference do also report a loss of body weight (or a lower rate of body weight gain), even where the CMS regime excludes food and water deprivation (e.g. Strekalova et al., 2011, Remus et al., 2015). Consequently, if CMS fails to change behaviour in the sucrose test, relative loss of body weight could be taken as an approximate proxy measure to inform a decision about whether to increase the intensity or variety of the CMS regime.

Another pointer to the importance of CMS intensity is a critical role of the hypothalamus-pituitary-adrenal (HPA) axis. The behavioural and physiological effects of CMS can be mimicked by chronic exogenous administration of corticosterone (Goshen et al., 2008, Gourley and Taylor, 2009, Kvarta et al., 2015), and blocked by the glucocorticoid receptor antagonist mifepristone (Wu et al., 2007), the corticosterone synthesis inhibitor metyrapone (Kvarta et al., 2015) or adrenalectomy (Goshen et al., 2008, Chen et al., 2016). These data strongly suggest that a chronically elevated physiological stress response, probably in the form of corticosterone spikes in response to the onset of each individual stressor (Sapolsky et al., 1984, Magariños and McEwen, 1995), is both necessary and sufficient for the behavioural effects of CMS – as discussed in more detail in the accompanying paper (Willner, 2016) and elsewhere (Willner et al., 2013).

### 4.3 The wider context

The wider context for the current study is the ongoing concern about reproducibility in biomedical research (Academy of Medical Sciences, 2015, Begley and Ioannidis, 2015, Steckler, 2015). This concern is driven by empirical studies demonstrating widespread failures to replicate published results. For example, failure rates of 75–90% were found for attempts within the pharmaceutical industry to replicate preclinical studies (Prinz et al., 2011, Begley and Ellis, 2012); similarly, a failure rate of around two thirds was reported for attempts to replicate experiments published in high ranking psychology journals (Open Science Collaboration, 2015). There has been extensive discussion of the reasons for this state of affairs. Problems with the original data include observer bias in non-blinded experiments, failure to repeat observations before publishing them, lack of legitimate controls, small sample sizes, use of inappropriate statistical tests, and selective publication of partial data sets (Begley, 2013, Button et al., 2013). Failures of replication often involve departures from the earlier protocol (Wahlsten, 2001, Wahlsten et al., 2003, Gilbert et al., 2016), but precise replication of experimental conditions may be of lesser importance than problems at source: for example, attempts to replicate published data in the fields of oncology, women's health and cardiovascular diseases found that some results were reproducible when using different protocols, while for others inconsistencies were found even when using an identical protocol (Prinz et al., 2011).

There is no clear consensus as to what constitutes a reproducible study because the inherent variability in biological systems means there is no expectation that results will necessarily be precisely replicated; rather, “the major conclusions that emerge from a scientific report should … withstand close interrogation” (Begley and Ioannidis, 2015). Steckler (2015) summarized this issue in the following terms: “A robust finding should be detectable under a variety of experimental conditions, making obsolete the requirement for exact, point-by-point reproduction. … (Most) replication studies are in fact studies testing the robustness of reported findings, since it may be difficult to exactly recapitulate all details and conditions under which the original data were produced. Moreover, robust data could be considered more important as they can be seen under varying conditions and may be biologically more relevant.”

Considering the overwhelming extent to which respondents to the present survey reported that they find the CMS model works reliably in their lab, and that every lab implements an idiosyncratic variant of the procedure, the conclusion that CMS causes a decrease in responsiveness to rewards and other depression-relevant changes in rodent behaviour should now be considered a highly robust finding. It might be of interest to conduct similar studies to evaluate the extent to which other animal models of psychiatric disorders are similarly robust.

### 4.4 Conclusions

This study has some significant limitations. In addition to those already discussed (the small size and diversity of the ‘less reliable’ sample of labs, the omission of labs that may have worked with the CMS model but not published their data, and the use of ad hoc measures to compare the severity of different stress regimes) are the use of survey methodology (which relies on opinion rather than hard data), the focus on the most widely used measures of CMS-induced anhedonia (to the exclusion of, for example, anxiety-like effects of CMS), and no discussion of the effectiveness of antidepressant treatments to reverse CMS effects (a topic that was touched on in the survey but in insufficient detail to justify reporting). Nevertheless, it is possible to draw two conclusions on the basis of the survey data and the published literature. First, the important take-home message is that the CMS model does appear to be generally reliable within laboratories and robust across laboratories; and the many published statements to the effect that the procedure is unreliable are incorrect. Second, while the critical features of the CMS procedure remain uncertain, success with using the model appears to depend on an interplay between (i) individual differences in susceptibility to stress, both within and between animal populations, (ii) the overall severity of the micro-stressors applied, which need to be sufficient intense to evoke a physiological stress response and sufficiently variable to prevent habituation to their repeated presentation, and (iii) good laboratory practices (for tips, see Strekalova and Steinbusch, 2010, Papp, 2012, Nollet et al., 2013), which are particularly important when the sucrose test is used as the main outcome measure.

## Figures and Tables

**Table 1:** Survey demographics.

	Number of labs identified				Survey requests		Responses	
	2010	2015	Total	% Overlap	Sent	%	Received	%
Europe	25	23	43	11.6	29	67	16	55
North America	11	29	39	2.6	34	87	25	74
China	33	93	118	6.8	76	65	13	17
Rest of World	12	36	46	4.3	31	67	17	55
Total	81	181	246	6.5	170	70	71	42

**Table 2:** Proportion of animals reported as ‘responding’ to CMS in relation to estimates of the overall reliability of the model.

	n	Proportion responding		
		25-50%	50-75%	>75%
Effects sometimes absent, or present but nonsignificant	9	2	6	1
Sucrose intake less reliable than other measures	6	0	4	2
CMS produces a reliable depressive phenotype	47	7	13	27

This table omits some labs that did not supply data on the proportion of responders, which includes the three labs where CMS was found to be ‘unreliable’.

**Table 3:** Characteristics of the CMS protocol in relation to estimates of its reliability.

	n	Duration	Variety	Severity	Burden
*A: Reliable?*					
Yes	51	22.7 (1.5)	7.2 (0.2)	0.36 (0.02)	20.7 (0.8)
Less so	14	26.0 (4.1)	7.7 (0.4)	0.32 (0.05)	21.8 (1.3)
*B: % responding*					
25-50%	8	21.8 (3.1)	6.8 (0.9)	0.35 (0.07)	19.5 (2.2)
50-75%	20	24.7 (2.8)	7.8 (0.3)	0.35 (0.04)	22.6 (1.1)
>75%	26	24.1 (2.4)	7.1 (0.3)	0.33 (0.03)	20.2 (1.0)

Duration: number of days of CMS; Variety: number of micro-stressors applied; Severity: proportion of micro-stressors rated 4 or 5 on the 5-point severity scale; Burden: total of all stress ratings. Values are mean (standard error). Six responses were excluded where respondents reported using both mice and rats, because it was uncertain whether the stress regime reported was applied to one species or to both. In B, there were additional omissions where the proportion responding was not reported.

**Table 4:** Most frequent components of the CMS regime.

Micro-stressor	% Overall	% Mice	% Rats	P
Wet bedding	79	83	71	
Cage tilt	75	**87**	**66**	0.049
Light-dark reversal	72	73	71	
Food deprivation	70	**57**	**86**	0.013
Water deprivation	65	**47**	**83**	0.004
Immobilization	52	**63**	**40**	0.083
Crowding	42	30	51	
Loud noise	39	40	37	
Stroboscopic lighting	38	30	46	
Forced swim	34	33	37	
Cold swim	28	30	23	

The most frequently used micro-stressors are shown overall and separately for mice and rats. The frequency of use of other micro-stressors was <20%. The right-hand column shows the results of Fisher's exact test for those micro-stressors where there was a significant or near-significant species difference (highlighted in bold).

**Table 5:** Outcome measures used, as a function of reported reliability.

	Group 1		Group 2		Groups 1 and 2		Group 3		Total (groups 1–3)	
	Less than reliable		Reliable x sucrose test				Reliable			
	n = 12	%	n = 6	%	n = 18	%	n = 53	%	N = 71	%
Sucrose/saccharin/sweet food intake/preference	7	58			7	39	23	45	30	42
Forced swim test			2	34	2	11	11	20	13	18
Anxiety (includes exploration tests and fear conditioning)	1	8	1	17	2	11	7	13	9	12
Grooming (includes coat state and splash test)			3	50	3	17	1	2	4	6
Multiple measures	3	25			3	17	1	2	4	6
Spatial cognition	1	8			1	6	3	7	4	6
Physiological measures							3	5	3	4
Alcohol intake							2	4	2	3
Other							2	4	2	3
